# Prediction of the composition of urinary calculi using artificial intelligence

**DOI:** 10.12669/pjms.41.7.11360

**Published:** 2025-07

**Authors:** Dan Shen, Tianxiong Yang, Tao Ma, Wenzeng Yang, Hongmei Li, Zhenyu Cui

**Affiliations:** 1 Dan Shen Department of Urology, Affiliated Hospital of Hebei University, Baoding 071000, Hebei, China; 2 Tianxiong Yang College of Clinical Medicine, Affiliated Hospital of Hebei University, Baoding 071000, Hebei, China; 3 Tao Ma Department of Urology, Affiliated Hospital of Hebei University, Baoding 071000, Hebei, China; 4 Wenzeng Yang Department of Urology, Affiliated Hospital of Hebei University, Baoding 071000, Hebei, China; 5 Hongmei Li Department of Urology, Affiliated Hospital of Hebei University, Baoding 071000, Hebei, China; 6 Zhenyu Cui Department of Urology, Affiliated Hospital of Hebei University, Baoding 071000, Hebei, China

**Keywords:** Calculi Composition, Faster R-CNN, Urinary Calculi

## Abstract

**Objective:**

To explore the capability and clinical application potential of the Faster Region-based Convolutional Neural Network (Faster R-CNN), an Artificial intelligence algorithm, in identifying the composition of urinary calculi from CT images.

**Method:**

This was a retrospective study. Data from 776 patients with urinary calculi treated at the Affiliated Hospital of Hebei University from August 2020 to December 2023 were collected. Patients with simple calculi were randomly divided into a model construction group and validation Group-I at a 5:1 ratio, while 60 cases of mixed calculi were randomly selected to form validation Group-II. The model construction group was employed to construct and test the performance of the Faster R-CNN model, while the validation groups were used to verify the model’s performance.

**Results:**

In validation Group-I, the model achieved an area under the curve (AUC) of 0.843. In validation Group-II, the kappa values for the model’s prediction of calcium oxalate and uric acid components, consistent with infrared spectroscopy analysis, were 0.649 and 0.653, respectively.

**Conclusion:**

Faster R-CNN demonstrates a robust capability for quantitative prediction of the composition of urinary calculi, indicating substantial promise for clinical applications.

## INTRODUCTION

Urolithiasis is a prevalent condition in urology, with an incidence rate ranging from 1% to 20%^1^ and a recurrence rate of 50% within five years.^2^-^3^ Urinary calculi can be categorized into calcium oxalate, calcium phosphate, magnesium ammonium phosphate, uric acid, cystine, and mixed calculi. Identifying the composition of calculi is crucial for guiding treatment decisions. For instance, patients with uric acid or cystine calculi may opt for medical treatment^4^, whereas monohydrate calcium oxalate calculi, cystine calculi, and carbonate apatite calculi, due to their high hardness, are less responsive to extracorporeal shock wave lithotripsy (ESWL).^5^ Non-invasive preoperative analysis of the composition of urinary calculi is of great reference value for clarifying the etiology, formulating treatment plans, and reducing recurrence rates. Currently, the infrared spectroscopy analysis method is commonly used in clinical practice for analyzing calculi composition, which has a lag in detection, as it requires obtaining calculi specimens.^6^ Object detection, a primary focus of artificial intelligence in medical research, involves locating and classifying objects within images.^7^ Faster R-CNN is a classic end-to-end target detection model.^8^ This study investigated the use of Faster R-CNN to predict the composition of urinary calculi, preliminarily exploring its capability to identify the composition of urinary calculi from CT images and its clinical application potential.

## METHODS

This study retrospectively analyzed CT images and calculi composition data from 776 patients treated at the Affiliated Hospital of Hebei University between August 2020 and December 2023, with preoperative CT images (free from artifacts, DJ stents, etc.) and postoperative infrared spectroscopy analysis reports. The dataset included 358 cases of simple calculi and 418 cases of mixed calculi. When collecting patient CT images, the first and last images were excluded to eliminate errors caused by the fusion of calculi with other body tissues; the calculi areas in CT images were marked as regions of interest (ROI) using the labeling tool LABELME. When collecting calculi infrared spectroscopy analysis reports, calculi were classified into simple calcium oxalate, calcium phosphate, magnesium ammonium phosphate, uric acid, cystine calculi, and mixed calculi based on quantitative data in the reports.

### Ethical approval:

The study was approved by the Institutional Ethics Committee of Affiliated Hospital of Hebei University (No.: HDFYLL-KY-2023-161; date: September 25, 2023), and written informed consent was obtained from all participants.

Patients with simple calculi were randomly divided into a model construction group and model validation Group-I at a 5:1 ratio using a random number method. The model construction group comprised 300 patients, who were further divided into training and test sets to construct and test the model’s performance; model validation Group-I consisted of 58 patients to validate model performance on simple calculi samples. An additional 60 cases from mixed calculi were randomly selected to form model validation Group-II using the random number method, which was used to validate the model’s performance on mixed calculi samples. Validation Group-II (60 mixed calculi) was designed to evaluate the model’s ability to detect calcium oxalate and uric acid components within heterogeneous samples, reflecting clinical complexity. Stratified sampling ensured proportional representation of subtypes during training.

### Target classification and dataset construction:

The model construction group comprised 300 cases of simple calculi patients, with a total of 7191 CT images. These included 5425 images of 251 cases of calcium oxalate calculi (5325 images of monohydrate calcium oxalate from 244 cases and 100 images of dihydrate calcium oxalate from 7 cases), 1488 images of 39 cases of uric acid calculi (1108 images of anhydrous uric acid from 28 cases, 375 images of dihydrate uric acid from 10 cases, and five images of monohydrate uric acid ammonium from one case), 200 images of 7 cases of calcium phosphate calculi, 10 images of one case of magnesium ammonium phosphate calculi, and 68 images of two cases of cystine calculi. Considering the small number of images for calcium phosphate, magnesium ammonium phosphate, and cystine calculi, to mitigate model classification accuracy issues due to data volume, a three-category model of calcium oxalate - uric acid - others (calcium phosphate, magnesium ammonium phosphate, and cystine) and a two-category model of calcium oxalate - uric acid were constructed.

The performance parameters of the two models were compared, and the model with better performance was selected for validation. Due to the varying number of calculi images among different patients, to achieve better training effects, training and test set samples were combined, and images were randomly divided at a 4:1 ratio. Each CT image of simple calculi patients was paired with its corresponding calculi composition to generate XML format files, and the training and test sets were prepared in the VOC2007 format dataset.

### Training the Faster R-CNN model:

The experimental environment was set up on the Ubuntu18.04 system, with experiments conducted under Python3.7, Pytorch1.2, and Cuda10.1 environment, leveraging NVIDIA GeForce RTX 2070 GPU, and Pycharm as the IDE tool. The calculi CT images were in BMP format, with a resolution of 512×512 pixels.

**Fig.1:**
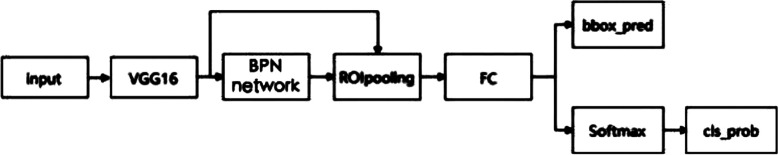
Faster R-CNN Network Structure Diagram.

**Fig.2:**
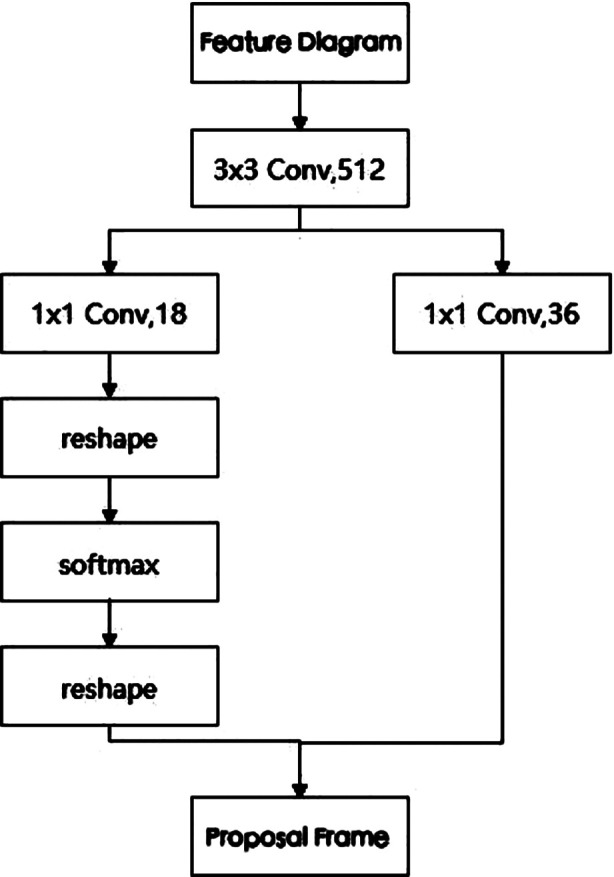
RPN Network Structure Diagram.

The model was trained based on the classic Faster R-CNN model network architecture (as shown in Fig.1): The prepared dataset was fed into the Faster R-CNN detection network, where image features were extracted and feature maps generated through the Vgg16 network. These feature maps were then input into the Region Proposal Network (RPN). The RPN network structure is shown in Fig.2, taking nine candidate boxes at each anchor point on the feature map (comprising three sizes and three aspect ratios), and determining whether the candidate box contains foreground or background, i.e., whether the candidate box contains the target. The network also calculated the deviation between the candidate boxes containing the foreground and the real target for preliminary boundary regression to obtain accurate proposal boxes. Subsequently, the feature map and proposal boxes were input together into the ROI pooling module to extract the feature map of the proposal boxes and fix it to the same size. The target category was ultimately output through the fully connected layer (FC) and prediction module. Model parameters were optimized using gradient descent, with a batch size of one, an initial learning rate of 0.001, a weight decay coefficient of 0.0005, and a momentum of 0.9, undergoing 30 epochs of training. Additionally, boundary regression loss was employed to adjust the position coordinates of the foreground target area, and classification loss was used to discard negative samples with confidence below 0.5. The network parameters were iteratively refined to achieve optimal results.

### Validation of the Faster R-CNN model:

The calculi images from validation groups one and two were input into the Faster R-CNN model to calculate its accuracy, sensitivity, specificity, positive predictive value, and negative predictive value in the validation groups. The number of accurately and inaccurately predicted images for each patient in validation Group-I was calculated to determine the model’s prediction accuracy. The model’s Receiver Operating Characteristic curve (ROC) was prepared, and its Area Under the Curve (AUC) was calculated to analyze the model’s diagnostic efficacy. In validation Group-II, a consistency test was conducted between the Faster R-CNN model and infrared spectroscopy analysis, with kappa values representing consistency.

## RESULTS

**Fig 3:**
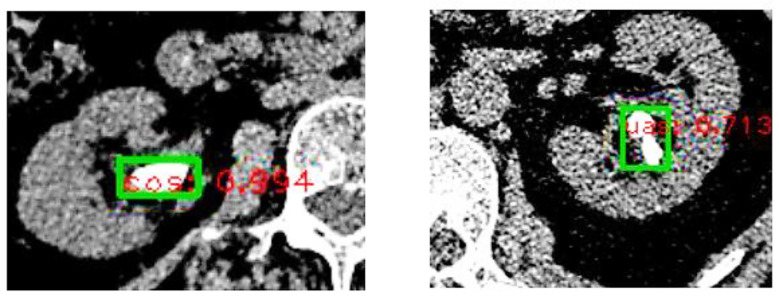
Example of Calculi Composition Prediction (cos stands for calcium oxalate, uas for uric acid, with numbers indicating probabilities).

The performance parameters of the training and test sets for the calcium oxalate - uric acid - other triple classification model are shown in Table-I. The performance parameters of the training and test sets for the calcium oxalate - uric acid binary classification model are shown in Table-II. The binary classification model demonstrated superior performance compared to the triple classification model, prompting further validation of the binary classification model. Examples of calculi composition predictions, along with associated probabilities, are illustrated in Fig.3. Validation set Group-I comprised 58 cases of simple calculi, with a total of 1,242 images. Of these, 50 cases (1,047 images) were identified as calcium oxalate (47 cases of monohydrate calcium oxalate with 943 images, three cases of dihydrate calcium oxalate with 104 images), and eight cases (195 images) were uric acid (seven cases of dihydrate uric acid with 188 images, one case of anhydrous uric acid with seven images). The performance parameters of the Faster R-CNN model for simple calculi are summarized in Table-III.

**Table-I:** Performance Parameters of the Triple Classification Model.

Dataset	Calculi Composition	Image Count (n)	Recall Rate (R, %)	Average Precision (AP, %)	Mean Average Precision (mAP, %)
Training Set	Calcium Oxalate	4340	90.66	85.33	75.95
	Uric Acid	1190	87.27	77.62	
	Other	222	80.00	64.91	
Test Set	Calcium Oxalate	1085	90.28	84.30	74.92
	Uric Acid	298	86.80	77.01	
	Other	56	79.10	63.44	

**Table-II:** Performance Parameters of the Binary Classification Model.

Dataset	Calculi Composition	Image Count (n)	Recall Rate (R, %)	Average Precision (AP, %)	Mean Average Precision (mAP, %)
Training Set	Calcium Oxalate	4340	91.69	89.68	85.03
	Uric Acid	1190	88.67	80.37	
Test Set	Calcium Oxalate	1085	91.06	89.32	84.67
	Uric Acid	298	88.20	80.03	

**Table-III:** Performance Parameters of the Faster R-CNN Model on Simple Calculi.

Calculi Composition	Image Count (n)	Sensitivity (%)	Specificity (%)	Positive Predictive Value (%)	Negative Predictive Value (%)	Accuracy (%)
Calcium Oxalate	1047	90.54	62.56	92.58	55.20	86.98
Uric Acid	195	62.56	90.54	55.20	92.58	

**Fig.4:**
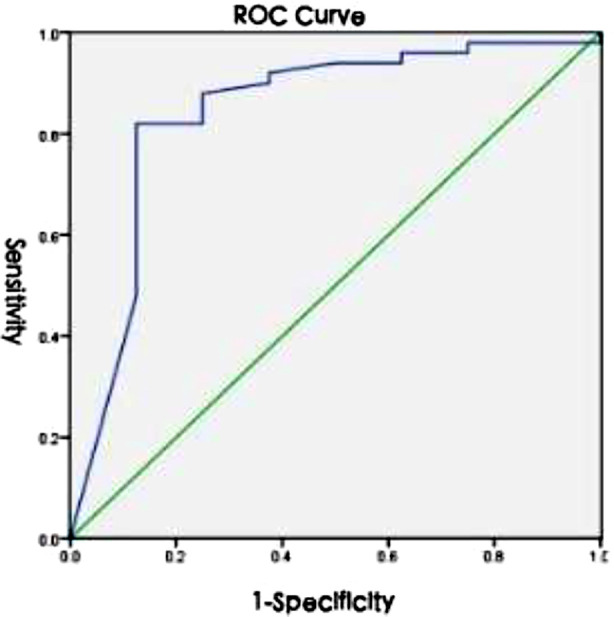
ROC Curve of the Faster R-CNN Model on Simple Calculi.

The prediction accuracy for each patient in validation Group-I was calculated by determining the number of correctly and incorrectly predicted images, and further generating the ROC curve, as shown in Fig.4. The area under the curve (AUC) was found to be 0.843 (95% CI: 0.668-1). Mixed calculi consist not only of calcium oxalate and uric acid but also of other components such as carbonate apatite. Thus, binary classification validation was performed to assess the presence or absence of calcium oxalate and uric acid. The performance parameters of the model on mixed stone samples are presented in Table-IV.

**Table-IV:** Performance Parameters of the Faster R-CNN Model on Mixed Calculi

Calculi Composition	Accuracy (%)	Sensitivity (%)	Specificity (%)	Positive Predictive Value (%)	Negative Predictive Value (%)	Kappa Value	95%CI
Calcium Oxalate	96.67	98.25	66.67	98.25	66.67	0.649	0.20, 1
Uric Acid	86.67	100	83.67	57.89	100	0.653	0.44, 0.86

## DISCUSSION

This study introduces the application of Faster R-CNN, a deep learning-based object detection framework, for in vivo prediction of urinary calculi composition using routine clinical CT images. The ability to preoperatively predict stone composition non-invasively has direct implications for clinical decision-making. For example, identifying uric acid components (sensitivity = 100% in mixed calculi) could enable targeted alkalization therapy. Moreover, bacterial toxins are often released during the lithotripsy process, leading to postoperative infections and even septic shock. Therefore, appropriate preoperative and postoperative measures are necessary to treat these calculi.^9^

Therefore, determining the composition of calculi in patients before treatment is crucial for identifying the underlying cause, formulating treatment strategies, and preventing recurrence. Infrared spectroscopy, the gold standard for calculi composition analysis, is hindered by inherent delays, and methods for preoperative calculi composition analysis are still under investigation. Clinically, ultrasound and CT are commonly used to diagnose urinary calculi. Some researchers have employed statistical methods to analyze ultrasound data^10^, CT values^11^, and dual-energy CT^12^ to predict calculi composition. However, these methods often lack sufficient accuracy and comprehensiveness. Artificial intelligence (AI), designed to simulate, extend, and augment human intelligence^13^, focuses on machine learning and deep learning algorithms.^14^

AI techniques are capable of efficiently analyzing and learning from different types of data, and have been widely used in various fields. Several scholars have applied AI to predict stone composition. Kriegshauser et al.^15^, Grosse et al.^16^, and Fitri et al.^17^ used AI algorithms to analyze CT images of calculi and predict calculi types with high accuracy. Among these, the deep learning algorithm CNN, used in Fitri et al.’s study^17^, achieved an accuracy rate of 99.6% in predicting calcium oxalate, uric acid, and mixed calculi. AI-based studies on in vitro calculi suggest that different types of calculi exhibit distinct characteristics on CT images, and these can be learned and classified by AI. However, research on in vivo calculi remains limited, with studies such as Kazemi et al.^18^ and Zhang et al.^19^ applying machine learning algorithms to predict calculi composition using in vivo CT images, though their results were less accurate and comprehensive.

Object detection represents a pivotal area of research within AI, focusing on the identification and classification of targets within images. The CNN algorithm, which has demonstrated superior performance in ex vivo studies, efficiently extracts image features through convolution operations. It has been applied to object detection, leading to the development of end-to-end target detection models that integrate candidate region generation, feature extraction, target classification, and location regression. A classic end-to-end model is the Faster R-CNN, introduced by Ren et al.^20^ The primary objective of this study is to employ CNN for the classification of calculi in CT images, aligning with the scope of object detection research. Consequently, the Faster R-CNN model, a classic end-to-end architecture in target detection, was employed in this study.

Due to insufficient data on other types of calculi (calcium phosphate, magnesium ammonium phosphate, and cystine calculi), models were constructed for a triple classification of calcium oxalate, uric acid, and other calculi, as well as a binary classification model for calcium oxalate and uric acid. The model’s performance was compared using the mean average precision (mAP) to select the more effective model for further validation. The findings indicated that the binary classification model, with a mAP of 84.67%, surpassed the triple classification model, which had a mAP of 74.92%, highlighting the impact of data volume on model accuracy. The binary classification model’s efficacy was further validated using simple and mixed calculi in verification groups one and two. In the validation Group-I, the AUC value was 0.843, and in the validation Group-II, the kappa values for predicting calcium oxalate and uric acid components using the model and infrared spectroscopy were 0.649 and 0.653, respectively, indicating that the model had high predictive performance in both simple and mixed calculi cases.

However, we also observed differences in sensitivity, specificity, positive predictive value, and negative predictive value when predicting calcium oxalate and uric acid calculi in both simple and mixed calculi. These differences may be attributed to three factors:

The volume of data for each stone type: In our training and testing datasets, we utilized data from 251 cases of calcium oxalate calculi, totaling 5425 images, and data from 39 cases of uric acid calculi, totaling 1488 images. The volume of data can significantly impact the model’s feature extraction precision and, consequently, the classification accuracy of different calculi compositions.

For instance, the Average Precision (AP) for other categories in the ternary model was substantially lower than that for calcium oxalate and uric acid; (2) the distribution of subtypes of calcium oxalate and uric acid calculi in the training and test datasets: The training and testing datasets included subcategories of calcium oxalate calculi, with 244 cases of monohydrate calcium oxalate totaling 5325 images, and 7 cases of dihydrate calcium oxalate totaling 100 images. The uric acid calculi subcategories included 28 cases of anhydrous uric acid totaling 1108 images, 10 cases of dihydrate uric acid totaling 375 images, and 1 case of monohydrate uric acid ammonium totaling five images. In validation Group-I, the subcategories of calcium oxalate calculi included 47 cases of monohydrate calcium oxalate totaling 943 images, and three cases of dihydrate calcium oxalate totaling 104 images.

The uric acid calculi subcategories included one case of anhydrous uric acid totaling seven images, and seven cases of dihydrate uric acid totaling 188 images. The volume of data for each subcategory in the training and testing datasets determined the precision of features incorporated into the model for each subcategory. When there is a significant difference between the subcategories in validation Group-I and those in the training and testing datasets, the prediction accuracy of the samples in validation Group-I may decrease; and (3) the limitations of Faster R-CNN: The Faster R-CNN algorithm loses network translation invariance due to two rounds of rounding approximations in the Region of Interest (ROI) Pooling operation, leading to a mismatch between detection information and extracted features. Additionally, its anchor box mechanism involves multiple down-sampling operations, which can impair the model’s detection effectiveness for small targets.^5^

### Limitations:

However, as a single-center retrospective study with a small and unevenly distributed sample size, this study has its limitations. The uneven distribution of mixed calculi subtypes (e.g., limited cystine or magnesium ammonium phosphate cases) may restrict generalizability to rare compositions. Future studies will incorporate multicenter data and advanced balancing techniques. The conclusions drawn here require further validation through prospective, large-scale, randomized controlled, and multicenter studies.

## CONCLUSIONS

This study is a preliminary exploratory research. Faster R-CNN demonstrates a robust capability for quantitative prediction of the composition of urinary calculi, indicating substantial promise for clinical applications.

### Author’s Contributions:

**DS** and **TY:** Conceived designed the study and Review. Was involved in preparing the manuscript

**TM, WY and HL:** Collected the data. Performed the analysis and Review.

**ZC:** Involved in the writing of the manuscript and is responsible for the integrity of the study.

All authors have read and approved the final manuscript.
